# Biomarkers and Immune Repertoire Metrics Identified by Peripheral Blood Transcriptomic Sequencing Reveal the Pathogenesis of COVID-19

**DOI:** 10.3389/fimmu.2021.677025

**Published:** 2021-08-24

**Authors:** Yang Liu, Yankang Wu, Bing Liu, Youpeng Zhang, Dan San, Yu Chen, Yu Zhou, Long Yu, Haihong Zeng, Yun Zhou, Fuxiang Zhou, Heng Yang, Lei Yin, Yafei Huang

**Affiliations:** ^1^State Key Laboratory of Virology, College of Life Sciences, Wuhan University, Wuhan, China; ^2^Department of Respiratory and Critical Care Medicine, Zhongnan Hospital of Wuhan University, Wuhan, China; ^3^Tongji Medical College, Huazhong University of Science and Technology, Wuhan, China; ^4^Center for Systems Medicine, Institute of Basic Medical Sciences, Chinese Academy of Medical Sciences & Peking Union Medical College, Beijing, China

**Keywords:** SARS-CoV-2, transcriptomic characteristics, IgG, machine learning, biomarker

## Abstract

The coronavirus disease 2019 (COVID-19) pandemic caused by severe acute respiratory syndrome coronavirus 2 (SARS-CoV-2) infection is a global crisis; however, our current understanding of the host immune response to SARS-CoV-2 infection remains limited. Herein, we performed RNA sequencing using peripheral blood from acute and convalescent patients and interrogated the dynamic changes of adaptive immune response to SARS-CoV-2 infection over time. Our results revealed numerous alterations in these cohorts in terms of gene expression profiles and the features of immune repertoire. Moreover, a machine learning method was developed and resulted in the identification of five independent biomarkers and a collection of biomarkers that could accurately differentiate and predict the development of COVID-19. Interestingly, the increased expression of one of these biomarkers, *UCHL1*, a molecule related to nervous system damage, was associated with the clustering of severe symptoms. Importantly, analyses on immune repertoire metrics revealed the distinct kinetics of T-cell and B-cell responses to SARS-CoV-2 infection, with B-cell response plateaued in the acute phase and declined thereafter, whereas T-cell response can be maintained for up to 6 months post-infection onset and T-cell clonality was positively correlated with the serum level of anti-SARS-CoV-2 IgG. Together, the significantly altered genes or biomarkers, as well as the abnormally high levels of B-cell response in acute infection, may contribute to the pathogenesis of COVID-19 through mediating inflammation and immune responses, whereas prolonged T-cell response in the convalescents might help these patients in preventing reinfection. Thus, our findings could provide insight into the underlying molecular mechanism of host immune response to COVID-19 and facilitate the development of novel therapeutic strategies and effective vaccines.

## Introduction

The outbreak of coronavirus disease 2019 (COVID-19), first reported in the city of Wuhan, China, in December 2019, has posed formidable threat to global public health (1–3). Owing to the rapid development of molecular virology, a new type of highly infectious coronavirus, officially named as severe acute respiratory syndrome coronavirus 2 (SARS-CoV-2), was quickly identified to be the pathogen of COVID-19 by deep sequencing and etiological analysis (4). SARS-CoV-2 replicates throughout the respiratory tract and results in both upper and lower respiratory tract infection including pneumonia (2, 5, 6). The virus preferentially invades the lung epithelial cells *via* angiotensin-converting enzyme 2 (ACE2) receptor (7–10) and might also enter other epithelial cells, immune cells, or other cell types through CD147 (BSG), CD26 (DPP4), or unidentified pathways (7, 11, 12). The clinical manifestations after SARS-CoV-2 infection are heterogeneous between individuals, ranging from asymptomatic, development of multiple symptoms such as coughing and fever, to failure of the respiratory system, and even death (13–15). Generally, this infection results in mild illness in most of the cases; however, approximately 15% of the patients need hospitalization, while nearly 5% of the patients develop acute respiratory syndrome and have high mortality, especially in the elderly and patients with underlying diseases (16). Therefore, there is an urgent need to develop novel therapeutic agents and effective vaccines for controlling the current pandemic, which requires the elucidation of the pathogenesis of COVID-19.

Recent investigations suggested that in addition to the virus itself, dysregulated host immune response to SARS-CoV-2 may also contribute to the pathogenesis of COVID-19 (17–20). The latter notion was supported by the facts that lymphophenia as well as increased number of neutrophils and monocyte were frequently observed in COVID-19 patients with severe illness (21–23). Along this line, several biomarkers were identified to be related to the disease courses of COVID-19, including excessive production of cytokines/chemokine (i.e., cytokine storm), delayed or aberrant type I interferon (IFN-I) responses, and other markers indicative of dysregulated immune response (24, 25).

Recently, researchers have used RNA-seq or single-cell RNA-seq (scRNA-seq) techniques to obtain transcriptomic data from COVID-19 patients in different disease courses, by which multiple biomarkers indicative of cell functions and immune response could be simultaneously determined at the transcriptional level. By these means, elevated expression of several proinflammatory cytokines including IL-6, IL-1β, MCP-1, IP-10, TNF, granulocyte colony-stimulating factor (G-CSF), and a weakened type I interferon (IFN-I) responses were reported in the peripheral blood of severe COVID-19 patients (21–23, 26). In addition, increased expression of biomarkers related to cell apoptosis and migration were also noted, especially in patients with severe illness. However, the dynamic changes of these biomarkers during the clinical course of COVID-19, which could provide insight into the contribution of immune response to COVID-19, are poorly characterized. Herein, we analyzed the host immune response to SARS-CoV-2 infection by profiling the quantitative transcriptomes using peripheral blood samples collected from 62 recovered COVID-19 patients, and published transcriptomic data of 16 acute patients and 17 healthy subjects downloaded from published papers/repository. Our analyses revealed that several biomarkers indicative of host immunity to SARS-CoV-2 infection, particularly the repertoire constitution of B cells and T cells, were substantially altered at the transcriptional level across different disease courses. To further identify the potential biomarkers that can accurately distinguish between different disease courses, a machine learning-based method was developed, by which five independent biomarkers and a sort of biomarker combination were successfully identified and validated. Moreover, the association between aforementioned biomarkers and the serum levels of SARS-CoV-2-specific antibodies across different disease courses was uncovered by correlative analyses. In addition, we also found that the level of *UCHL1*, a marker for brain damage, was significantly upregulated in acute patients compared with that in healthy donors and convalescent patients.

## Materials and Methods

### Ethics Approval

The work was performed in line with the Declaration of Helsinki principles for ethical study. The study was approved by the Medical Ethics Committee of the Tongji Medical College, Huazhong University of Science and Technology, Wuhan, China [Clinical Ethical Approval No. (2020) IEC-A251].

### RNA-Seq Data Analysis

The RNA-seq data of acute and healthy control were retrieved from the NCBI-GEO dataset, with ID PRJNA639275 (27). RNA-seq raw data were cleaned using Fastp (v0.20.1) and then mapped to human genome (GRCh38) using HISAT2 (v2.2.0). FeatureCounts tool of Subread package (v2.0.0) was used for counting the number of reads aligned to genes, with the help of GENECODE gene annotation (v34). Then analysis of differentially expressed genes was performed with DESeq2 R package (v1.30.0). Genes were categorized as differentially expressed with the criteria: adjusted *p*-value (pAdj) <0.05 and |log2FoldChange| >1 for upregulated genes and the adjusted *p*-value <0.05 and |log2FoldChange| <−1. Gene counts were normalized by DESeq2 and converted to *log_2_* (*normalized counts* + 1) format. Then batch effect was removed by limma R package (v3.46.0) and the result was taken as the normalized gene expression level.

### GO and KEGG Enrichment Analysis

GO and KEGG enrichment analyses of differentially expressed genes (DEGs) were performed using clusterProfiler R package (3.18.0). The Benjamini and Hochberg method was used to adjusted *p*-value. Significantly enriched GO term and KEGG pathway were selected under the criteria: adjusted *p*-value <0.05.

### Calculation of B-Cell Receptor and T-Cell Receptor Repertoire Metrics

We applied MiXCR (v.3.0.12) to RNA-seq data from peripheral blood mononuclear cells (PBMCs) for reconstructing B-cell receptor (BCR) and T-cell receptor (TCR) reads. To minimize batch effects and address the unevenness of sequencing depth, sequencing data volume (the number of reads multiplied by the length of reads) was kept at the same level. The targeted sequencing of the TCR beta chain CDR3 to distinguish T-cell clones, frequency, and antigenicity is feasible in TCR repertoire identification (28, 29).

BCR and TCR clonotypes were extracted using MiXCR (v.3.0.12). “Analyze shotgun” command was used with parameters “–impute-germline-on-export” and “–only-productive”. The post-analysis of BCR/TCR repertoire was conducted using VDJtools (v1.2.1).

The number of unique clonotypes per sample was used to indicate richness. Unique clonotype was strictly defined by exact match for receptor nucleotide sequence (CDR3 sequence, V/J segments, and hypermutations). Shannon entropy was used to represent diversity. Shannon entropy is expressed as:

$$H=-\sum_{i=1}^{N}p_{xi}.ln(p_{xi})$$

Where *N* represents the total number of clonotypes in sample X and *p_xi_* refers to the frequency of clonotype *i*. Generally speaking, more unique clonotypes and a more even distribution of clonotypes will increase the diversity of a given repertoire.

1-Pielou’ evenness was used to represent clonality which ranges from 0 to 1, and zero clonality means that each clonotype has the same frequency. Higher clonality often means that there is a group of highly expanded clonotypes with dominant frequencies.

Pielou’ evenness is expressed as:

$$E=\frac{H}{H_{max}}$$

Where *H* represents Shannon entropy and *H_max_* is calculated as ln(*N*). (*N* represents the total number of clonotypes in a sample).

The clonality is expressed as:

Clonality=1−E

In CDR3 length analysis, the samples in each group were analyzed all together. Length distribution was described by cumulative distribution function (CDF).

### Prediction of COVID-19 Outcomes Based on Machine Learning

We used a logistic regression model as the classifier. The logistic regression function is expressed as follows:

$$logit=b_{0}+b_{1}f_{1}+b_{2}f_{2}+\ldots +b_{n}f_{n}$$

Where *f*_1_ to *f_n_* represent different features. *b*_0_ indicates bias and *b*_1_ to *b_n_* denote the weight of these features. A sigmoid function was applied to transform logit into a score ranging between 0 and 1. The sigmoid function is expressed as follows:

$$score=\frac{1}{1+e^{-logit}}$$

As a Python module integrating classical machine learning algorithms, Sklearn (v0.23.1) was used to implement our model. To evaluate the performance achieved by each combination of features, a three-fold cross-validation was performed. The samples were randomly split into three sets and each set was called a fold. For each fold, it was taken as a test set and the remaining two folds were treated as a training set. Then, this progress was repeated until all folds had served as the test set. The performance of a model was characterized by the mean accuracy and mean AUC of results tested on each fold. For each combination, a ROC curve was outputted according to the result of the prediction. The average AUC of the curves was compared and the final output combination was determined with the highest AUC.

### Enzyme-Linked Immunosorbent Assay

SARS-CoV2 S1 protein (Sino Biological Inc., BJ) was coated on a high-binding 96-well plate (Thermo Scientific) at 4°C overnight. Plates were blocked with 5% non-fat milk in phosphate-buffered saline (PBS) for 1 h at room temperature, followed by incubation with 1:100 diluted plasmas in dilution buffer (PBS, 2% non-fat milk, and 0.05% Tween-20). A 1:4,000 dilution of horseradish peroxidase (HRP)-conjugated mouse anti-human IgG, IgM, and IgA antibodies (BaiaoTong Experiment Center, LY) was added and incubated for 1 h at room temperature. Wells were washed six times between each step with 0.05% Tween-20 in PBS (PBST). Finally, wells were developed using tetramethylbenzidine substrate (Beyotime Inc., WH) and were stopped by the addition of stop solution, followed by reading at 450 and 570 nm. The sample OD was calculated as OD450 subtracted by OD570.

### Statistical Analysis

Statistical analyses were performed with GraphPad Prism software (Version 6.0, GraphPad Software Inc.). Throughout the study, *n* refers to the number of subjects where every subject is one data point. Unpaired two-group comparisons were done with the Mann–Whitney *U* test. In figures, ****P < 0.0001, ***P < 0.001, **P < 0.01, and *P < 0.05.

## Results

### Study Subjects and Study Design

A total of 62 COVID-19 convalescents, 16 acute patients, and 17 healthy donors were involved in this study. The information of subjects including age, sex, disease severity, and time from symptom onset or a negative RT-PCR test to sample collection is summarized in **Table 1**. Of note, the samples of healthy controls were collected prior to the outbreak of COVID-19. Acute patients with COVID-19 were all hospitalized, while 60 of the 62 convalescents were hospitalized. The percentages of hospitalized patients in the acute infection group and convalescent group were 100% and 96.7%, respectively. The median time from symptom onset to sample collection for acute patients was 14 days, with an interquartile range of 9–16 days. In the convalescent group, the median time from a negative RT-PCR test to sample collection was 151.0 days, with an interquartile range of 135–162 days. The majority of these convalescents (51 in 62; 82.2%) were moderate cases, and only 2 were mild cases, and 9 were severe cases (**Table 1**).

**Table 1:** List of information of the patients.

	Convalescent group (*n* = 62)	Acute group (*n* = 16)	Healthy group (*n* = 17)
Age, years	55.0 (49.3–63.0)	52.5 (39.3–68.0)	43.0 (36.0–55.0)
Gender			
Female	47 (75.8%)	9 (56%)	9 (53%)
Male	15 (24.2%)	7 (44%)	8 (47%)
Time from symptom onset to sampling, days	NA	14 (9–16)	NA
Time from the last negative test to sampling, days	151.0 (135. 0–162.0)	NA	NA
Disease severity status			
ICU	0	4 (25%)	No
Severe	9 (14.5%)	8 (50%)	No
Moderate	51 (82.2%)	4 (25%)	No
Mild	2 (3.3%)	0	No
Underlying diseases			
None	35 (56.5%)	NA	NA
Hypertension	18 (29.0%)	NA	NA
Diabetes	11 (17.7%)	NA	NA
Coronary heart disease	2 (3.2%)	NA	NA
Hyperlipidemia	2 (3.2%)	NA	NA
Rheumatoid arthritis	2 (3.2%)	NA	NA
Hepatitis B	2 (3.2%)	NA	NA
Tuberculosis	1 (1.6%)	NA	NA
Nephritis	1 (1.6%)	NA	NA
Urticaria	1 (1.6%)	NA	NA
Breast cancer	1 (1.6%)	NA	NA

Data are median (IQR), n (%), or n/N (%). P-values were calculated by the Mann–Whitney U test, χ² test, or Fisher’s exact test, as appropriate.

NA, not available.

**Figure 1:**
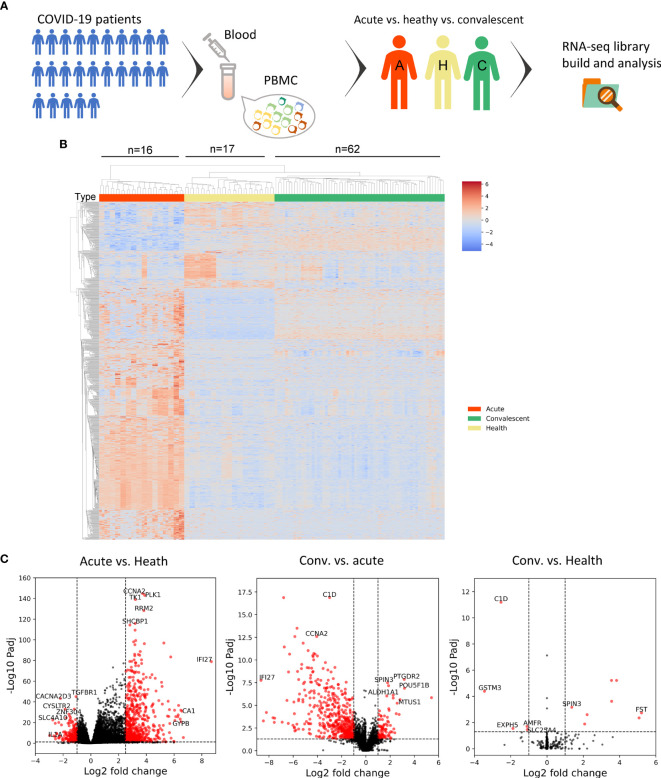
Transcriptomic analysis shows the alteration of host immunity in acute and convalescent SARS-CoV-2 infection. **(A)** Design of the experiment. PBMC was prepared from patients, while total RNA was extracted and analyzed to identify differentially expressed genes (DEGs). **(B)** DEGs across acute COVID-19 patients (*n* = 16), healthy donors (*n* = 17), and convalescents (*n* = 62). **(C)** Volcano plot on DEGs in the comparison of different groups. Each red dot represents an individual gene with Benjamini–Hochberg adjusted *P*-value (two-sided unpaired Wald test) ≤ 0.01 and average log2 fold change ≥ 0.5 in the comparison.

Ficoll density gradient centrifugation was performed to isolate the PBMCs, followed by the construction of RNA library and high-throughput sequencing (**Figure 1A**). Quality control was applied for the RNA-seq data, and batch effect was minimized by including a pooled mixture of all samples as standard control.

### Transcriptomic Profiling of PBMCs From Acute COVID-19 Patients, Convalescents, and Healthy Donors

In order to investigate the peripheral alteration during and after SARS-CoV-2 infection, we compared the transcriptomic differences in PBMC samples from acute COVID-19 patients, convalescents, and healthy donors through DEG analysis. The combined DEG list is shown in the **Supplementary Data**.

The heat map of the DEG profiles was built (**Figure 1B**), through which the profile of acute COVID-19 patients was found to be clearly different from that of convalescents and health donors (HDs). Of note, the DEG profile of convalescents was comparable to that of HD, suggesting that the overall gene expression pattern of acute patients was significantly altered compared with that of HDs, and almost returned to the normal level of healthy individuals in the convalescent phase.

To further study the transcriptomic changes induced by SARS-CoV-2 infection regarding the specific cellular biological processes, we performed a volcano plot (**Figure 1C**) followed by gene functional enrichment analysis (**Figure S1**) to identify differentially expressed genes and enriched pathways.

In the comparison between acute COVID-19 patients and HDs, 668 genes were upregulated and 230 genes were downregulated in acute COVID-19 patients. Whereas in the comparison between convalescents and acute patients, 475 DEGs were downregulated in convalescents, compared with 91 genes that were upregulated. When the DEGs of convalescents and HDs were compared, sparse DEGs were noted and the fold change of gene expression was not evident, indicating the resemblance between these two cohorts from the transcriptomic perspective.

Interestingly, the upregulated genes in acute patients compared with those in HDs were enriched in multiple biological processes, which were also found to be downregulated in convalescents compared with those in acute patients, as revealed by GO term and KEGG pathway enrichment analysis (**Figure S1**). Of note, the viral infection-induced alterations in PBMCs of convalescents were enriched in gene modules including “complement activation,” “humoral immune responses,” “phagocytosis,” and “adaptive immunity based on somatic recombination.” In addition, the upregulation of a series of B-cell-related gene modules was also observed, including “immunoglobulin receptor binding” and “antigen binding.” These findings suggested that the expression of genes related to humoral immunity may be upregulated in the acute phase of SARS-CoV-2 infection, thereafter declining and finally returning to the ground level in convalescents. To conclude, our data provided a quantitative and global insight into the dynamic changes of immune-related genes, such as those implicated in B-cell response during SARS-CoV-2 infection, especially in the convalescent phase.

### Immune Repertoire Analysis Reveals the Increase of B-Cell Diversity and Richness in Acute Patients

**Figure 2:**
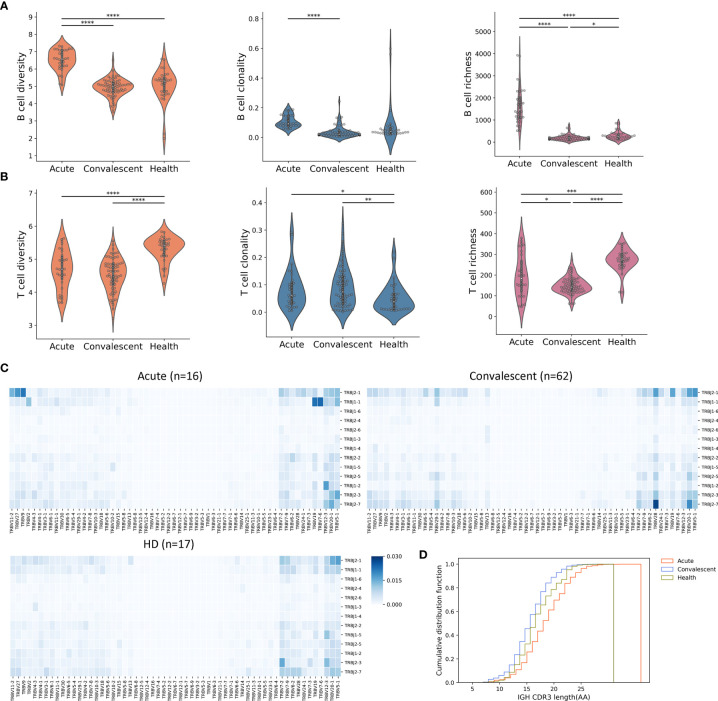
Peripheral B-cell and T-cell diversity, richness, and clonality analyzed by RNA-seq. **(A)** The B-cell repertoire was characterized by diversity, richness, and clonality, as calculated on the basis of BCR sequence similarity including CDR3 sequence, as well as IGHV and IGHJ usage. *P < 0.05, **P < 0.01, ***P < 0.001, ****P < 0.0001. **(B)** Characterization of peripheral T cells. **(C)** Heat maps showing T-cell V(D)J rearrangement in PBMC across three cohorts. The colors suggested the frequency of a particular V-J gene pair. **(D)** CDR3 length comparison of IGH. The acute patients seemed to have the maximum IGH CDR3 length, indicating frequent BCR recombination.

To study the alteration of the B-cell and T-cell repertoire during and after SARS-CoV-2 infection, we retrieved the complementarity determining region 3 (CDR3) variable region of the beta chain of the TCR and heavy chain of the BCR from the RNA-seq library. B-cell diversity, as estimated by unique CDR3 usage, was significantly higher in acute COVID-19 patients compared with that in convalescents (*P* < 0.0001) and HDs (*P* < 0.0001) (**Figure 2A**). Next, richness, a measurement used to quantify the number of unique V(D)J rearrangements, was evaluated and also found to be significantly higher in acute COVID-19 patients than in convalescents and HDs. Finally, clonality, an indicator used to characterize clonal amplification and represents the evenness of the repertoire, was higher in acute patients than in healthy controls and recovered people. To better explain the meaning for quantification of B-cell diversity, richness, and clonality, a toy model was used and displayed in **Figure S2**. In sum, the highest clonality, diversity, and richness of B-cell repertoire were observed in acute COVID-19 patients, highlighting a biased and uneven B-cell repertoire developed in peripheral blood during the acute phase of SARS-CoV-2 infection, which may reveal the dynamic nature of B-cell response during SARS-CoV-2 infection.

The overall T-cell metrics for these subjects were also calculated according to the CDR3 sequence of the beta chain of the TCRs (TRB). Notably, the highest level of TRB diversity was observed in HDs (**Figure 2B**), whereas the TRB diversity of acute COVID-19 patients and convalescents was significantly lower than that of HDs (*P* < 0.0001, *t*-test). However, the acute patients and convalescents had significant higher T-cell clonality than HDs, indicating the clonal expansion of antigen-specific T cells and the shift of T-cell repertoire. The richness of TRB sequence, which represents the distribution of unique clonotypes, showed a similar trend as the diversity. Together, lower diversity and evenness as well as higher clonality of T cells in acute and convalescent patients may indicate the durability of T-cell response to SARS-CoV-2 infection.

We next sought to compare the profiles of TCR gene usage between the three groups. In this regard, significantly distinct profiles were observed in TRB genes (**Figure 2C**), but were less pronounced in TRA (alpha chain of the TCRs) genes (**Figure S3**). An overrepresentation of TRBJ2-7 was identified in convalescents, whereas an overrepresentation of TRBJ1-1 and TRBJ2-1 was observed in acute patients. Interestingly, we also discovered TRBV28/TRBJ2-7 as the top V-J pair in convalescents. The distinct usage of V(D)J genes between acute patients and convalescents could be explained by a repertoire shift reflecting the transition from acute T-cell response to memory T-cell response.

Previous studies had shown that TCR structural difference, such as CDR3 length, might contribute to the differentiation of T cells (30). To test this issue, we analyzed the CDR3 length of BCR and TCR in the three cohorts and found that B cells from acute patients tended to have a longer IGH (heavy chain of immunoglobulin) CDR3, whereas no differences were observed in T cells (**Figure 2D** and **Figure S4**), suggesting that heterogeneous BCR recombination might be induced mainly in IGH upon SARS-CoV-2 infection.

Taken together, these results highlighted the complex and imbalanced nature of host immune responses to SARS-CoV-2 infection, in which B-cell response may be substantially induced in the acute phase and return to the ground level in the convalescent phase, whereas T-cell response could persist for a long time even without the presence of specific antigen in the convalescent phase.

### Machine Learning Models to Classify COVID-19 Cases and Predict Clinical Outcomes

Next, a computational pipeline was developed based on the transcriptomic data of our cohort to identify the potential biomarker or biomarker combination that could accurately distinguish between different disease courses of COVID-19. More importantly, we aimed to distinguish the convalescents from the unexposed HDs, since some convalescents who were asymptomatic could not be successfully identified by nucleic acid testing (NAT) or antibody testing.

**Figure 3:**
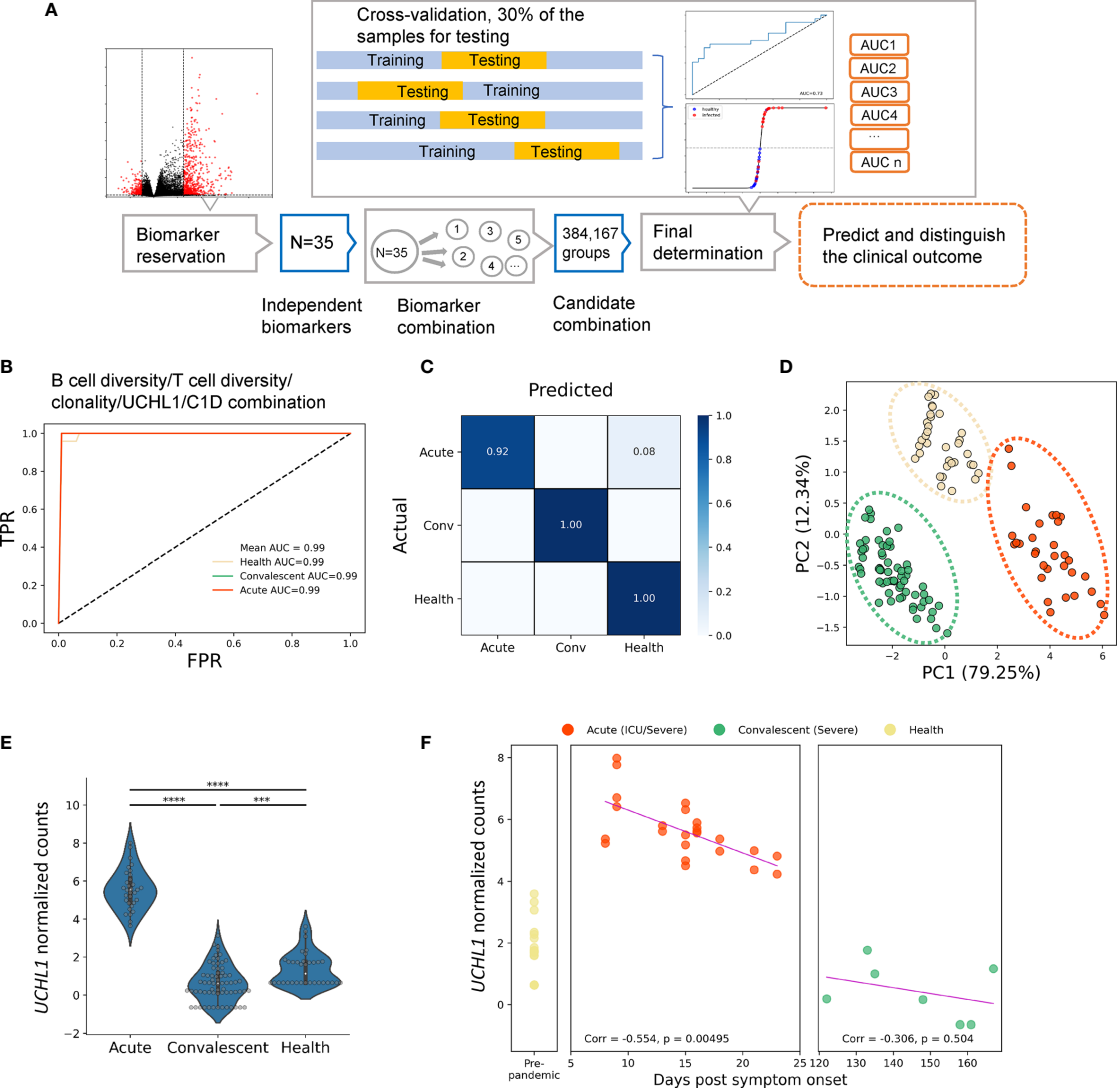
Identification of biomarkers for classifying and predicting clinical outcomes of SARS-CoV-2 infection by machine learning strategy. **(A)** The experiment design of the prediction pipeline; cross-validation was involved for model training. DEG, differentially expressed genes. The best biomarker combination determined by the pipeline: **(B)** the ROC curves, **(C)** the confusion matrix, and **(D)** PCA analysis for the best biomarker combination, which was more accurate and sensitive than independent biomarkers. **(E)** Normalized UCHL1 expression in acute, convalescent, and healthy groups. **(F)** Correlation between normalized values of UCHL1 expression and time from symptom onset to sample collection. The pink line represents the linear regression line. Unpaired two-group comparisons were done with the Mann–Whitney U test. ****P < 0.0001, ***P < 0.001.

Firstly, all transcriptomic data were normalized on indexes including repertoire metrics and DEGs before machine learning algorithm was applied. Twenty-nine biomarkers consisting of 20 repertoire metrics and 9 highly ranked DEGs chosen from 1,241 DEGs were taken as candidate biomarkers to generate about 146,595 groups of candidate biomarker combinations. Samples were randomly divided into two groups, with 70% and 30% of the samples allocated to the training set and the testing set, respectively. Cross-validation was preformed using these datasets to determine the preferred biomarker combination with the highest area under the curve (AUC) value (31–33). For the model training in this step, multivariate logistic regression (LR), a widely accepted machine learning algorithm, was performed (**Figure 3A**). This approach resulted in the identification of five independent biomarkers which could be used to predict disease outcome and distinguish between acute patients, convalescents, and HDs (**Figure S3**). These biomarkers include the differentially expressed gene C1D nuclear receptor corepressor (*C1D*), Alpha-2-adrenergic receptors (*ADRA2A*), spindlin family member 3 (*SPIN3*), ubiquitin C-terminal hydrolase L1 (*UCHL1*), and Glutathione S-Transferase Mu 3 (*GSTM3*). To determine the reliability of these biomarkers, confusion matrices (**Figure 3C**) were built and showed that various diseases courses of these samples could be distinguished with ideal accuracy with these biomarkers. Moreover, a best biomarker combination was also determined, including B-cell diversity, T-cell diversity, and clonality, as well as the expression levels of *UCHL1* and *C1D*. This combination included both DEGs and repertoire features and thus resulted in optimal accuracy of the model. The AUC of acute, convalescent, and HD reached 0.99 in this model (**Figure 3B**). Moreover, the principal component analysis (PCA) (**Figure 3D**) demonstrated that the different clusters of samples were clearly segregated by this combination, further suggesting the reliability of this prediction model.

Thus, our findings demonstrated that the clinical outcome of a patient can be predicted by a combination of repertoire signatures and DEGs through machine learning approaches, thereby providing insight into the classification and prediction of COVID-19 development and invoking timely and precise clinical advice for the treatment of COVID-19.

### Potential Use of UCHL1 as Peripheral Blood Biomarker for Neurological Complications in COVID-19 Patients

Among the aforementioned biomarkers, *UCHL1* is particularly interesting given the following reasons: i) it is a biomarker frequently used for indicating brain damage induced by viral infections; ii) the encoded protein can be tested in the blood sample, making it clinically applicable; iii) it is one of the five “predictors” identified by our machine learning approach. Therefore, we next focused on this molecule. Firstly, we confirmed the normalized gene expression profiles of *UCHL1* measured by RNA-seq in each cohort with or without COVID-19 (**Figure 3E**). After normalization, the expression level of UCHL1 was still found to be significantly higher in acute patients than in convalescents and HDs.

Therefore, we next sought to explore the association of clinical outcomes with *UCHL1* levels at the time of sampling (**Figure 3F**). For patients with severe symptoms or admitted to the ICU, the *UCHL1* level was negatively associated with the time from symptom onset to sample collection (Corr = −0.554, *P* = 0.0049). A tendency of negative correlation was also noted when all acute patients were included for analysis; however, this correlation failed to reach statistical significance (Corr = −0.173, *P* = 0.344).

Together, we found that *UCHL1* expression used as a biomarker in the peripheral blood can predict the disease development of COVID-19, although the potential mechanism by which this molecule participates in the pathogenesis of COVID-19 requires further investigations.

To further elaborate the role of UCHL1 in COVID-19, we reviewed human studies investigating the use of serum UCHL1 as biomarker for brain injury. In a multicenter cohort study including 206 patients and 175 healthy controls, UCHL1 level was effective in discriminating between TBI (traumatic brain injuries) patients with and without intracranial lesions on a CT scan and was correlated with functional 3-month outcomes (34). In another study with 273 mild and moderate TBI patients, similar results were observed and the combination of GFAP and UCHL1 was sensitive for identifying a positive CT scan (35). Another observation cohort study on HIE (hypoxic–ischemic encephalopathy) patients (neonate patients = 16, controls = 11) also found that UCHL1 was elevated in HIE neonates and associated with cortical injury (36). As a protein biomarkers for central nervous system damage, UCHL1 has been detected in diverse brain injuries including ischemic/hemorrhagic stroke (37, 38), TBI (39, 40), Parkinson’s disease (41), cardiac arrest (42), and seizures (43).

The role of human coronaviruses in nervous system damage has been underestimated. Nevertheless, recent evidence suggests that the development of SARS-CoV-2 infection could induce neurological disorders involving both the central and peripheral nervous systems in COVID-19 patients worldwide (44–48); however, the pathogenesis and the sequelae of the damage are still poorly understood. Our transcriptional analysis suggests that the elevated serum UCHL1 protein levels could be a hallmark of severe illness in acute COVID-19 patients; theoretically, this protein might be involved in opportunistic direct attack on the brain or systemic secondary insult including thrombosis, hypoxemia, or autoimmune response. Therefore, future clinical investigations are warranted to build the connection of this molecule with severe illness of COVID-19 at the protein level given the accessibility of blood sample and the potential clinical relevance.

### Clonality of Peripheral T Cells in Convalescents Is Positively Correlated With the Serum Levels of Spike Protein-Specific IgG

**Figure 4:**
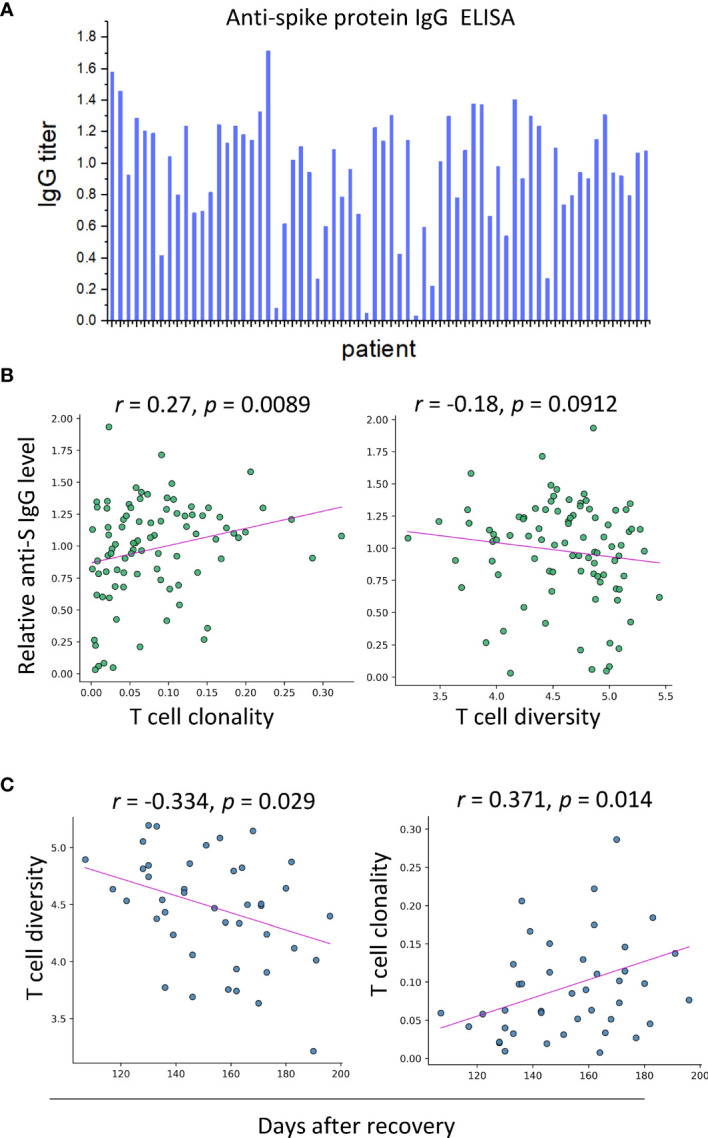
SARS-CoV-2-specific IgG is correlated with T-cell clonality in serum samples of convalescent patients (*n* = 62). **(A)** The results of ELISA assays. **(B)** Correlation between anti-S IgG level and T-cell clonality and T-cell diversity, respectively. **(C)** T-cell diversity of blood samples of convalescents is negatively correlated with days after recovery, and T-cell clonality exhibits an increasing trend against days after recovery.

The appearance of T-cell diversity in the final biomarker combination which can accurately predict disease outcomes suggests that the structure of T-cell repertoire might contribute to anti-virus response during SARS-CoV-2 infection. To validate this speculation, the S protein-specific IgG levels of blood samples of convalescent individuals (*n* = 62) were measured by enzyme-linked immunosorbent assay (ELISA) (**Figure 4A** and **Table S1**), and correlative analyses were performed to determine their relationship with the immune repertoire metrics extracted from transcriptional analysis. These analyses revealed that the S protein-specific IgG levels were found to be positively correlated with T-cell clonality and diversity (*r* = 0.27; *P* = 0.0089 and *r* = −0.18; *P* = 0.0912, Spearman rank correlation) (**Figure 4B**). It is noteworthy to mention that even from patients who had been discharged for more than 200 days, the S protein-specific IgG was still detectable with a relatively high titer, accompanied by the persistence of memory T-cell response. Surprisingly, the S protein-specific IgG levels were not associated with those B-cell repertoire metrics. Nonetheless, the lack of association between antibody levels and B-cell response has been reported by other groups, which requires further investigation (discussed later).

We next sought to determine the kinetics of the T-cell proliferation. For this purpose, time of sample collection was incorporated into our analysis. The T-cell diversity was negatively correlated with days after recovery (*r* = −0.334; *P* = 0.029, Pearson correlation) (**Figure 4C**). However, the clonality of T cells exhibited a growing trend over time (*r* = 0.371; *P* = 0.014, Pearson correlation). The opposite trends of diversity and clonality could be explained by the clonal expansion of specific types of T cells, suggesting the prolonged maturation of T-cell responses against SARS-CoV-2 infection.

To conclude, the correlation between T-cell expansion and the SARS-CoV-2-specific antibody responses highlighted the contribution of T cells to the humoral immune responses. Besides, the persistent presence of T-cell response and virus-specific IgG antibody supports the notion that immune memory was maintained in convalescent COVID-19 patients for up to 6 months.

## Discussion

Viral infection often causes dramatic alteration on the host transcriptome, which may contribute to the aberrant immune responses and facilitate the invasion of the virus (49–52). Using the PBMC specimens of 62 convalescents, 16 acute patients, and 17 health donors, we reported here the genome-wide transcriptomic analyses that characterize the host immune responses during SARS-CoV-2 infection at the transcriptional level. The major findings of this study can be summarized as follows: i) several biomarkers including UCHL1 and a combination of biomarkers were identified using machine learning-based method with great accuracy in predicting the clinical outcome of COVID-19; ii) the B-cell response declines to the normal level in convalescents, as shown by similar repertoire profiles between HDs and convalescents and the lower expression of genes involved in humoral immunity in HDs and convalescents compared with that in acute patients; and iii) SARS-CoV-2-specific antibody response and T-cell response can be detected in the convalescents who recovered for up to 6 months, suggesting the existence of prolonged immune protection against reinfection in these patients.

The differential expressed gene analysis found 898 DEGs from acute patients compared with healthy controls. These DEGs have been compared with those from other published analyses using RNA-seq or single-cell RNA-seq data (53–55). As shown in **Figure S6**, *SPARC*, *S100A12*, *ITGB3*, *LCN2*, *IL1B*, and *TNFAIP3* are found to be the shared response genes among these studies and ours. *SPARC* encodes a cysteine-rich acidic matrix-associated protein, which is also involved in extracellular matrix synthesis and promotion of changes to cell shape. *S100A12* relates to innate immune system and TLR4 signaling. *LCN2* also relates to innate immunity and IL-17 signaling pathway. *ITGB3* and *IL1B* both relate to ERK signaling. The expression of *TNFAIP3* is induced by TNF and is involved in the signaling pathway by GPCR. In conclusion, the most common genes shared by our and other DEG analyses are highly related to immune response and immune regulation.

These findings provided valuable perspectives for the pathogenesis of SARS-CoV-2 infection. B-cell diversity was significantly increased in acute patients compared with that in healthy subjects; thereafter, it gradually decreased over time in convalescents and finally declined to the levels comparable with that in healthy subjects (**Figure 2** and **Figure S7**). However, for T-cell diversity, a unanimous decreasing tendency was noted from acute phase to convalescent phase (**Figure S7**). The distinct kinetics of T- and B-cell repertoire across SARS-CoV-2 infection suggests that although B-cell response might subside over time after the initial activation in the acute phase, the response of T cell to SARS-CoV-2 could persist for a long time even without the presence of specific antigens.

The presence of SARS-CoV-2 spike protein-specific IgG antibodies was observed in long-term recovered individuals from COVID-19, which is supported by the recent findings that most convalescent patients still harbor high titers of neutralizing antibodies against SARS-CoV-2 in the study cohorts from the United States, Iceland, and China (56–60). Presumably, the first wave of antibody production might occur during acute SARS-CoV-2 infection and is likely mediated by short-lived plasmablasts which are capable of escaping the germinal center reaction and resulting in the production of immature antibodies with limited somatic hypermutation. This wave of antibodies may decline in 1 month due to the short half-lives of antibodies and plasmablasts. However, the second wave of antibody production by plasma cells is dominant afterwards. With experience in the germinal center (61–63), plasma cells are capable of producing mature antibodies with a higher frequency of somatic hypermutation and quickly moving to bone marrow where they can find survival niches and persistently secrete antibodies of high quality. The correlation of SARS-CoV-2 spike protein-specific IgG antibodies with T-cell repertoire metrics but not B-cell repertoire metrics is rather interesting. A similar finding was reported recently by Mathew et al. using a flow cytometry approach (21). Perhaps, prolonged T-cell response will provide additional help for either the antibody-producing cells or B cells outside the periphery, or only a subset of B cells in the periphery that was not identified in this study.

To identify biomarkers that can monitor or predict the progress of the disease is of high priority in fighting against the pandemic. We combined both repertoire metrics and 1,241 DEGs to profile the immune alteration in response to SARS-CoV-2 infection and identified 5 biomarkers through machine learning. Interestingly, these biomarkers could be individually used to distinguish COVID-19 outcomes, suggesting that the differential expression of these genes is implicated in the development of the disease. Moreover, a combination of biomarkers was identified with outstanding accuracy for predicting disease outcome. Strikingly, not only could this model be used to distinguish between the different phases of COVID-19 patients, it is also effective in distinguishing unexposed HDs from convalescents who have a negative antibody test result. This approach will improve the accuracy of identifying and monitoring SARS-CoV-2-infected individuals with undetectable antibody and virus RNA, which is beneficial for the screening and control of the pandemic.

However, the high level of signals generated by inflammation related to infection may sequester other signals that are also involved in the pathogenesis of COVID-19. One of these signals may be the pathways related to nervous system injury, both for the central nervous system and peripheral nervous system, partially due to their preferential distribution in the nervous system but not in peripheral blood. However, with emerging reports suggesting the presence of brain injury in some subset of COVID-19 patients, which is consistent with the previously reported effects of the coronavirus on human nervous system, there is an urgent need to identify peripheral biomarkers with the capacity to early identify neurological complications after SARS-CoV-2 infection. We reported here the association between the peripheral biomarker UCHL1 and severe disease of COVID-19. Serum UCHL1 has been a biomarker for brain damage in various studies including those complicated with virus infection. Thus, the role of this molecule in the nervous system should be further explored, especially in the context of COVID-19. Once the connection between UCHL1 and the neurological complication of COVID-19 is firmly established, the presence of this molecule could be used for the early diagnosis of neurological disorder caused by SARS-CoV-2 attack and favor the therapeutic intervention of COVID-19 patients.

In conclusion, our transcriptomic analyses involving peripheral samples from 62 convalescents, 16 acute patients, and 17 healthy controls uncovered the distinct kinetics of T-cell and B-cell response to SARS-CoV-2 infection, with B-cell response plateaued in the acute phase and declined thereafter, whereas T-cell response can be maintained for up to 6 months post-infection onset and may provide help to assist in the maintenance of anti-SARS-CoV-2 antibodies. Using a machine learning approach, five independent biomarkers, and a collection of multiple biomarkers including both DEGs and immune repertoire metrics, were identified to be capable of predicting disease outcome with great accuracy. One of these biomarkers, UCHL1, is of particular importance given its potential role in mediating nervous system complications after SARS-CoV-2 infection. Thus, our findings will lay the ground for the future exploration of the pathogenesis of COVID-19 and may facilitate the use of the aforementioned biomarkers in clinical diagnosis and intervention.

## Data Availability Statement

The raw data supporting the conclusions of this article will be made available by the authors, without undue reservation.

## Ethics Statement

The studies involving human participants were reviewed and approved by Medical Ethics Committee of the Tongji Medical College, Huazhong University of Science and Technology, Wuhan, China. The patients/participants provided their written informed consent to participate in this study.

## Author Contributions

LeY, YH, YuZ, and YC conceived and designed the study. YW, YL, and YouZ did the bioinformatic analyses. BL recruited the patients. BL and FZ provided the clinical information. HZ, DS, YunZ, HY, and LoY performed the experiments. YL wrote the initial draft of the manuscript. YH and LeY provided comments and helped edit the manuscript. All authors contributed to the article and approved the submitted version.

## Funding

This work was supported by the National Natural Science Foundation of China (31870728 and 31470738), the Science Foundation of Wuhan University (2042020kfxg02 and 2042016kf0169), Translational Medicine and Interdisciplinary Research Joint Fund of Zhongnan Hospital of Wuhan University (Grant No. ZNJC202006) to LeY, and the Innovative Foundation of Huazhong University of Science and Technology (3004510131) to YH.

## Conflict of Interest

The authors declare that the research was conducted in the absence of any commercial or financial relationships that could be construed as a potential conflict of interest.

## Publisher’s Note

All claims expressed in this article are solely those of the authors and do not necessarily represent those of their affiliated organizations, or those of the publisher, the editors and the reviewers. Any product that may be evaluated in this article, or claim that may be made by its manufacturer, is not guaranteed or endorsed by the publisher.
