# Supplementary material for: Biomarkers and Immune Repertoire Metrics Identified by Peripheral Blood Transcriptomic Sequencing Reveal the Pathogenesis of COVID-19
Source: Front Immunol. 2021 Aug 24;12:677025. doi: 10.3389/fimmu.2021.677025 (PMC8421539; doi:10.3389/fimmu.2021.677025)
Supplement: Supplementary file 3 [file DataSheet_3.pdf]

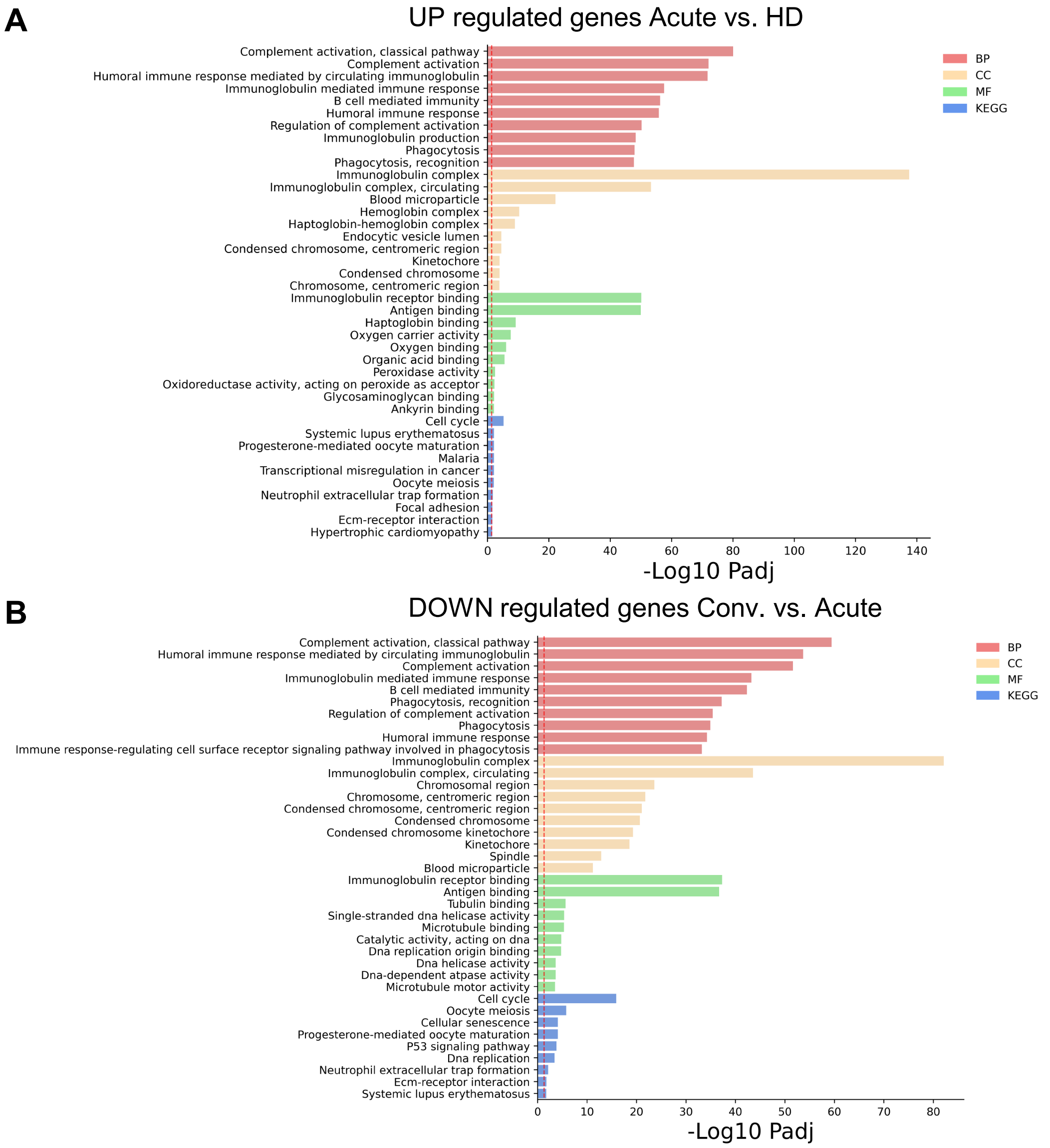

Figure S1. GO-term and KEGG pathway enrichment analysis of differentially expressed genes. (Related to Figure 1). (A) GO-term functional enrichment of up-regulated expressed genes in acute patients' PBMC versus HDs. GO-term functional enrichment includes 3 categories (biological process, BP, molecular function, MF, cellular component, CC). (B) Same as (A) for down-regulated genes in convalescent patients' PBMC versus acute patients'.

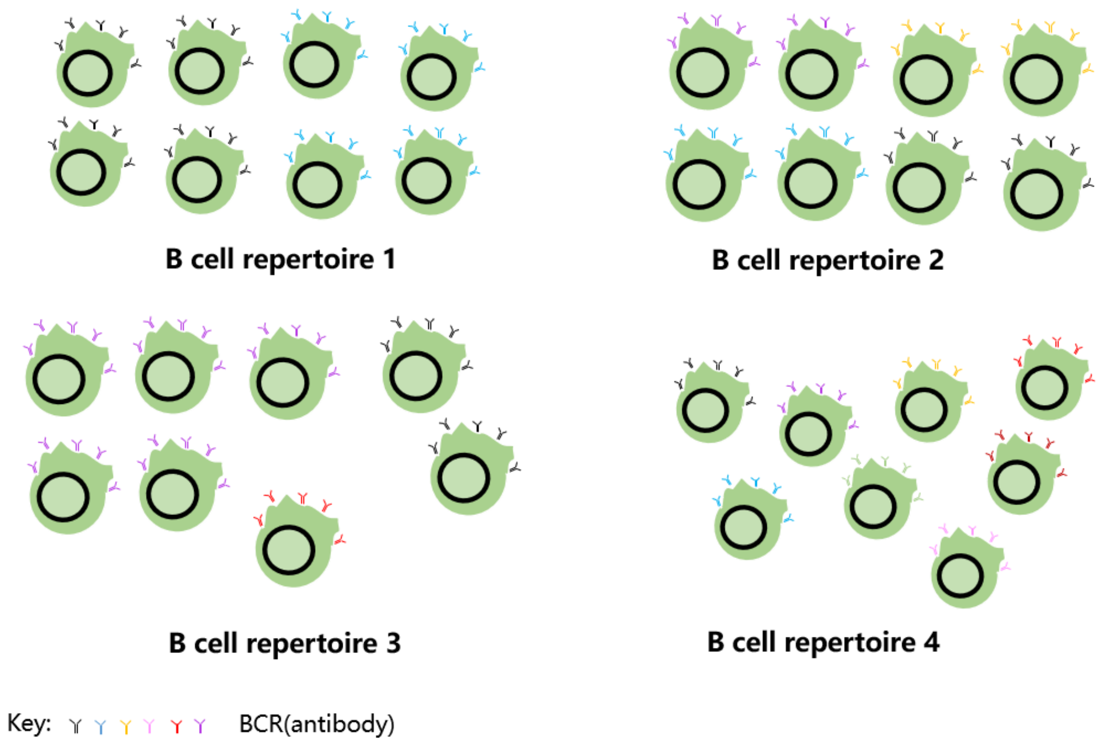

	B cell repertoire 1	B cell repertoire 2	B cell repertoire 3	B cell repertoire 4
Shannon entropy	0.693	1.386	0.900	2.079
Diversity	0.693	1.386	0.900	2.079
Richness	2	4	3	8
Clonality	0	0	0.181	0
Evenness	1	1	0.819	1

Figure S2. Toy models of B cell repertoire characterizaion. (Related to Figure 2). Each green circle represents a B cell and the color of Y on the circle represents a different BCR sequence.

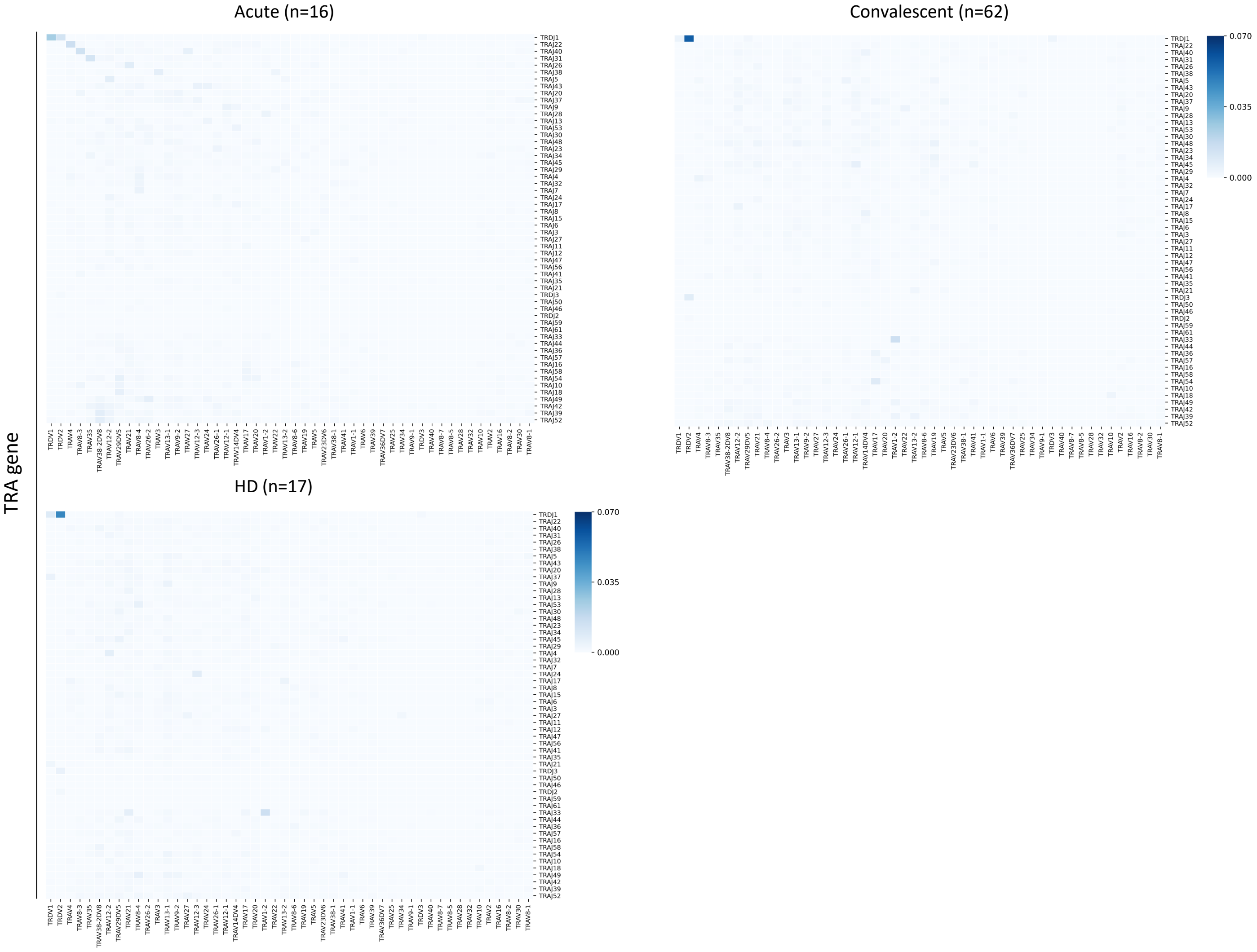

Figure S3. Data on T cell repertoire analysis. (Related to Figure 2C). V(D)J rearrangement in TRA genes across three conditions: acute infected, convalescent and Healthy donor.

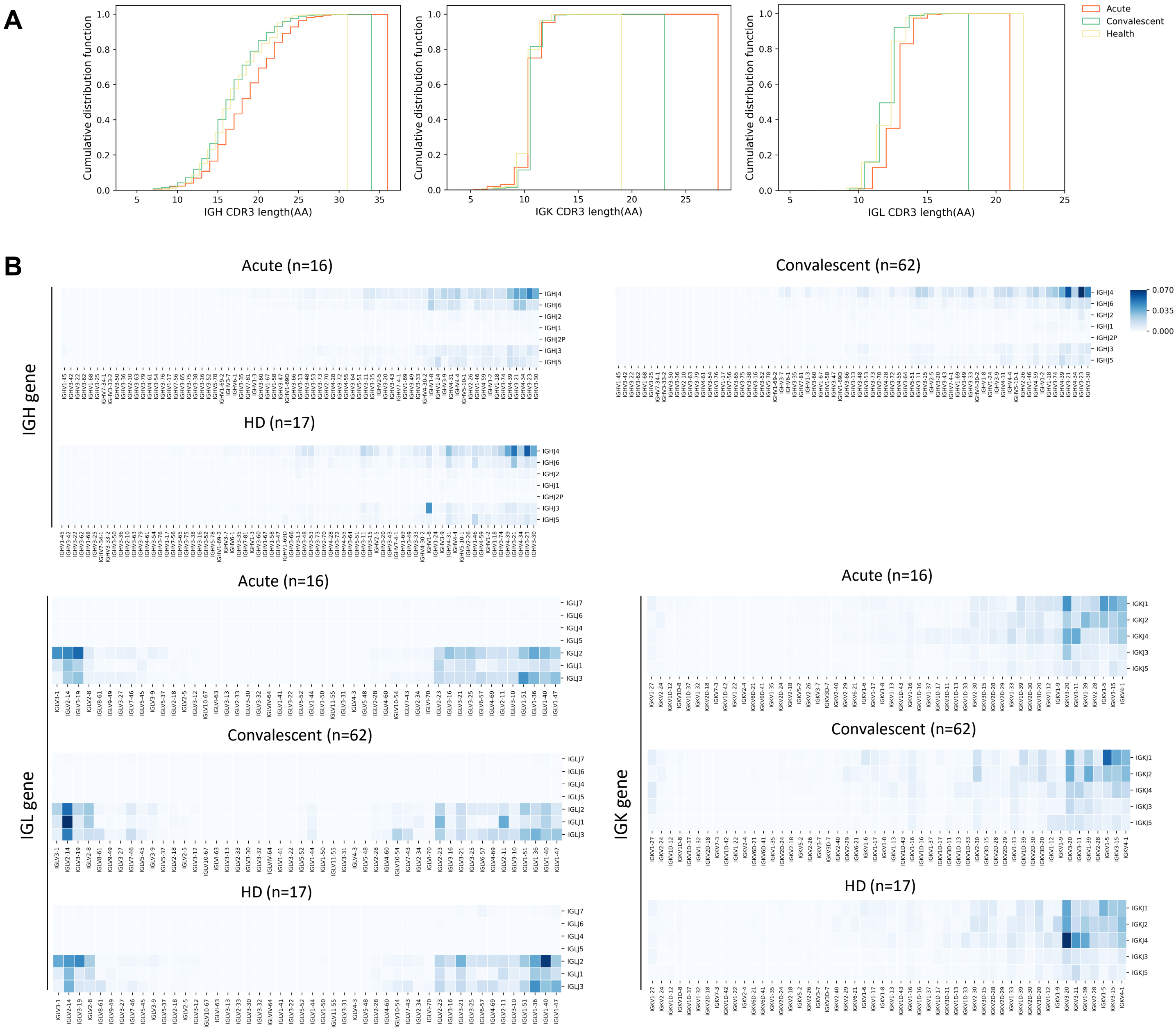

Figure S4. CDR3 length and selective usage of V(D)J genes analyses on B cells. (Related to Figure 2).
(A) CDR3 length of IGH, IGK, IGL segment of the B cells.
(B) Heat maps showing IGH, IGK, IGL V(D)J rearrangement in B cells across three cohorts. The colors suggest the frequency of a particular V-J gene pair.

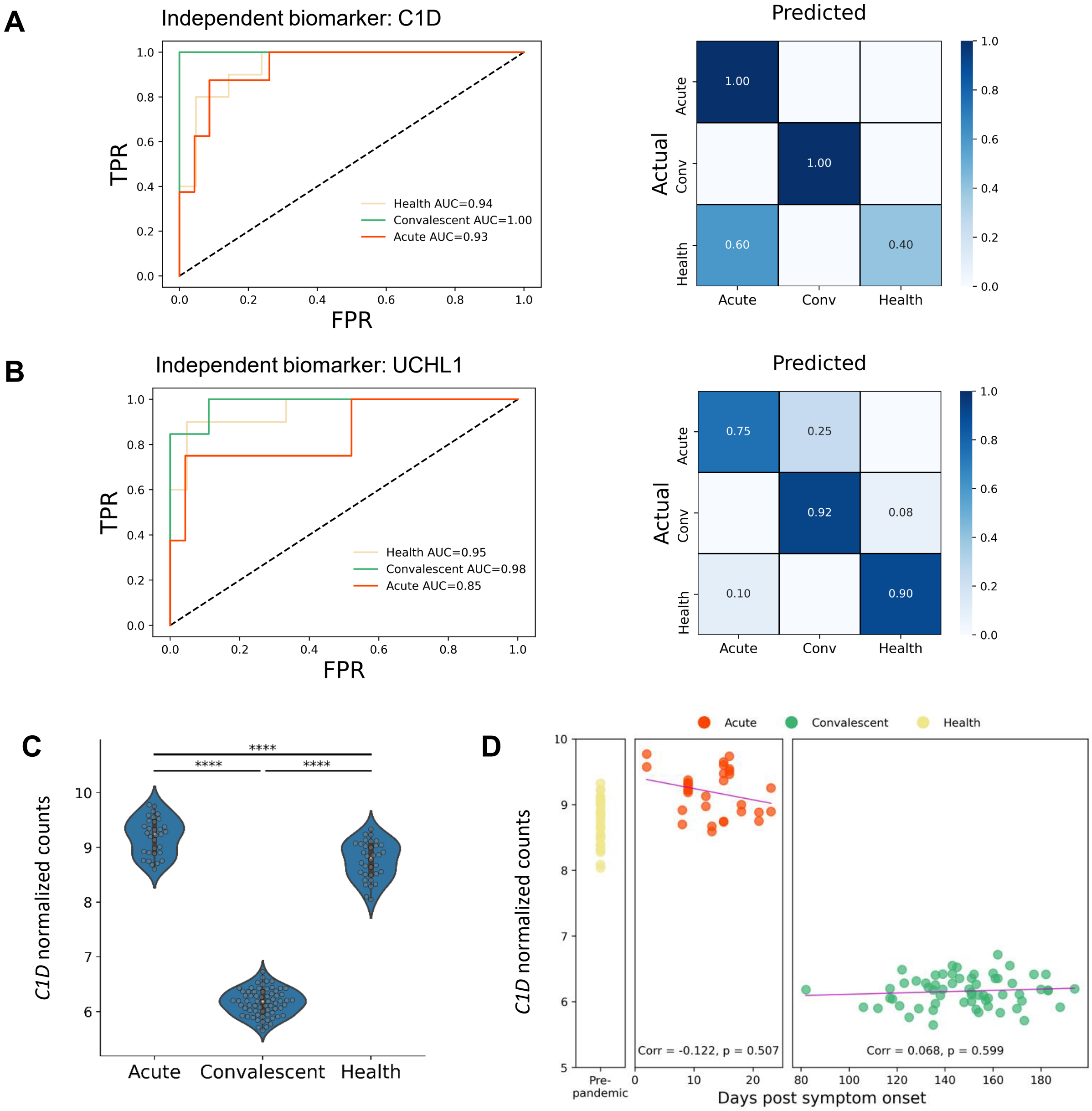

Figure S5. Determination of the biomarkers to accurately distinguish and predict clinical outcomes of SARS-CoV-2 infection by machine learning strategy. (Related to Figure 3).

(A) Receiver operating characteristic (ROC) curves and confusion matrix for an independent biomarker C1D determined by the machine learning pipeline. AUC values were calculated for the classification of HD, convalescent, moderate, severe and ICU COVID-19 patients, respectively. TPR: true positive rate; FPR: false positive rate; AUC: The area under the ROC curve.

(B) Same as (A) but for another independent biomarker UCHL1.

(C) Normalized C1D1 expression in three groups.

(D) Distribution of the C1D biomarker and association with clinical outcomes of patients with COVID-19

Group	Study	Method
DEG1	Alsamman M. Alsamman, Hatem Zayed et.al(53)	RNA-seq
DEG4 cohort1	Jonas Schulte-Schrepping, Nico Reusch et.al(54)	single-cell RNA-seq
DEG4 cohort2	Jonas Schulte-Schrepping, Nico Reusch et.al(54)	single-cell RNA-seq
DEG5	Aymeric Silvin, Nicolas Chapuis et.al(55)	single-cell RNA-seq
This research	Yang Liu, Bing Liu et.al	RNA-seq

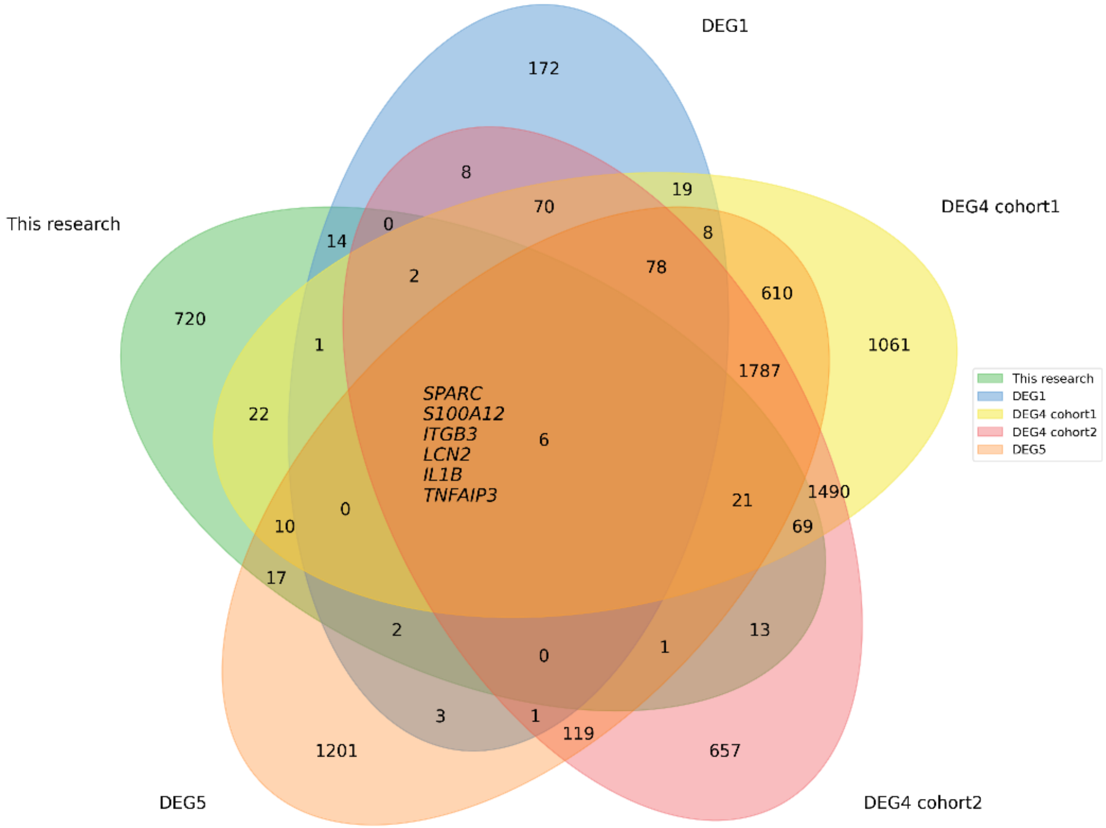

Figure S6. The Venn diagram of DEGs from independent studies. (Related to Figure 1). The number of uniquely shared DEGs related to host response induced by SARS-CoV-2 infection are labelled.

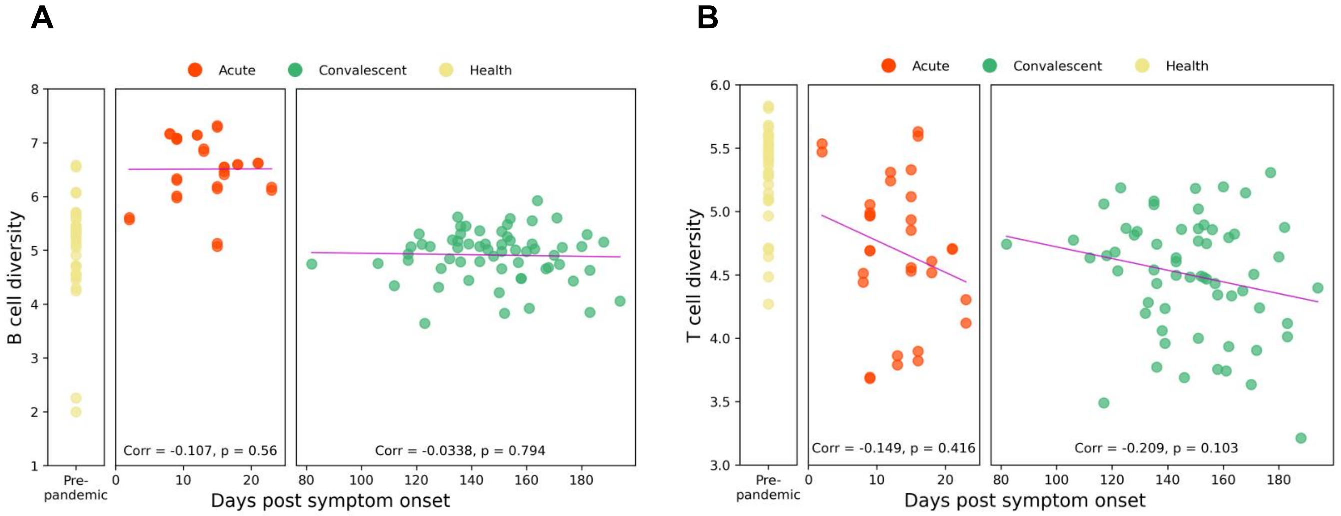

Figure S7. Changes of the diversity of B cells and T cells over time post-infection. (Related to Figure 2).

(A). The relationship between B cell diversity and days post infection are shown by scatter plots. The pink line represents the linear regression line.

(B). Similar as A but for T cell diversity.

.

Table S1

Sample_ID	Type	IgG titer to S protein	Inhibition rate	Age	Sex	Classification according to IgG
Conv029	Convalescent	1.5823	71.31%	41	male	High
Conv031	Convalescent	1.4578	60.24%	61	male	High
Conv033	Convalescent	0.9250	45.18%	48	female	Medium
Conv034	Convalescent	1.2879	53.63%	50	female	High
Conv035	Convalescent	1.2077	64.64%	65	female	Medium
Conv036	Convalescent	1.1916	64.29%	41	female	Medium
Conv037	Convalescent	0.4166	41.85%	36	male	Low
Conv038	Convalescent	1.0419	56.19%	50	female	Medium
Conv039	Convalescent	0.8011	51.13%	55	female	Medium
Conv040	Convalescent	1.2364	69.05%	49	female	Medium
Conv041	Convalescent	0.6851	68.94%	50	female	Low
Conv042	Convalescent	0.6949	70.83%	50	female	Low
Conv043	Convalescent	0.8161	73.64%	66	female	Medium
Conv044	Convalescent	1.2443	79.95%	59	female	High
Conv045	Convalescent	1.1298	70.74%	54	female	Medium
Conv046	Convalescent	1.2379	71.06%	41	male	Medium
Conv048	Convalescent	1.1814	75.02%	37	male	Medium
Conv049	Convalescent	1.1477	76.08%	31	female	Medium
Conv050	Convalescent	1.3285	87.28%	46	female	High
Conv051	Convalescent	1.7148	93.96%	43	female	High
Conv052	Convalescent	0.0829	59.68%	37	male	Low
Conv053	Convalescent	0.6187	58.89%	46	female	Low
Conv054	Convalescent	1.0231	68.71%	43	female	Medium
Conv055	Convalescent	1.1079	77.47%	41	female	Medium
Conv056	Convalescent	0.9420	72.67%	51	female	Medium
Conv057	Convalescent	0.2646	29.91%	40	male	Low
Conv058	Convalescent	0.6022	68.66%	46	female	Low
Conv059	Convalescent	1.0874	61.47%	51	female	Medium
Conv060	Convalescent	0.7849	64.75%	50	female	Low
Conv061	Convalescent	0.9645	36.77%	22	male	Medium
Conv063	Convalescent	0.6801	71.52%	33	male	Low
Conv064	Convalescent	0.0478	54.06%	28	male	Low
Conv065	Convalescent	1.2268	70.32%	54	female	Medium

Table S1. Data of patient information and ELISA assays. (Related to Figure 4).
The assay measures the SARS-CoV-2-S-responsed IgG and neutralizing efficacy of serum samples from recovered COVID-19 individuals.

Table S1-continue

Sample_ID	Type	IgG titer to S protein	Inhibition rate	Age	Sex	Classification according to IgG
Conv066	Convalescent	1.1415	69.82%	47	male	Medium
Conv068	Convalescent	1.3042	77.51%	47	female	High
Conv069	Convalescent	0.4266	59.77%	43	female	Low
Conv070	Convalescent	1.1497	72.58%	55	female	Medium
Conv071	Convalescent	0.0317	54.42%	unknown	unknown	Low
Conv072	Convalescent	0.5955	69.26%	unknown	unknown	Low
Conv073	Convalescent	0.2226	61.11%	unknown	unknown	Low
Conv074	Convalescent	1.0127	68.48%	39	male	Medium
Conv076	Convalescent	1.2994	76.50%	64	male	High
Conv077	Convalescent	0.7822	75.35%	50	female	Low
Conv078	Convalescent	1.0836	71.01%	55	female	Medium
Conv079	Convalescent	1.3782	84.10%	59	female	High
Conv080	Convalescent	1.3707	71.43%	57	female	High
Conv081	Convalescent	0.6644	67.14%	60	female	Low
Conv082	Convalescent	0.9779	75.94%	63	female	Medium
Conv083	Convalescent	0.5405	64.79%	51	female	Low
Conv084	Convalescent	1.4055	94.42%	51	female	High
Conv085	Convalescent	0.9053	65.85%	55	female	Medium
Conv087	Convalescent	1.3005	83.55%	56	female	High
Conv089	Convalescent	1.2374	84.19%	60	female	Medium
Conv090	Convalescent	0.2694	73.09%	45	female	Low
Conv091	Convalescent	1.0994	75.71%	69	female	Medium
Conv094	Convalescent	0.7373	68.20%	44	female	Low
Conv095	Convalescent	0.7937	66.91%	45	female	Low
Conv096	Convalescent	0.9424	63.50%	40	female	Medium
Conv097	Convalescent	0.9034	64.93%	30	female	Medium
Conv098	Convalescent	1.1531	90.83%	62	female	Medium
Conv102	Convalescent	1.3090	87.93%	68	female	High
Conv104	Convalescent	0.9405	74.88%	67	female	Medium
Conv105	Convalescent	0.9199	65.90%	63	female	Medium
Conv107	Convalescent	0.7945	73.32%	63	male	Medium
Conv114	Convalescent	1.0666	82.07%	59	female	Medium
Conv117	Convalescent	1.0782	66.73%	71	female	Medium
